# Supplementary material for: Peanut meal-based wood adhesives enhanced by urea and epichlorohydrin
Source: R Soc Open Sci. 2019 Nov 27;6(11):191154. doi: 10.1098/rsos.191154 (PMC6894569; doi:10.1098/rsos.191154)
Supplement: Supporting information 1 [file rsos191154supp1.doc]

**Preparation of three-ply plywood**

Three layer poplar veneers were interlaced vertically and horizontally, and the adhesive was applied on both sides of the middle poplar veneer. One-sided glue amount was 200 g/m^2^. Then, the three-layer poplar veneer was hot-pressed (1.0 MPa, 130°C, 600 s) using an XLB-350 (Qi Cai Hydraulic Machinery Co., Ltd., Shanghai, China). After the hot pressing, the plywood samples were laid at room temperature for 24 h, then cut into test-pieces.
